# Learning Visible Thermal Person Re-Identification via Spatial Dependence and Dual-Constraint Loss

**DOI:** 10.3390/e24040443

**Published:** 2022-03-23

**Authors:** Chuandong Wang, Chi Zhang, Yujian Feng, Yimu Ji, Jianyu Ding

**Affiliations:** School of Computer Science, Nanjing University of Posts and Telecommunications, Nanjing 210023, China; chdwang@njupt.edu.cn (C.W.); 1320047722@njupt.edu.cn (C.Z.); 2020070134@njupt.edu.cn (Y.F.); 1220044922@njupt.edu.cn (J.D.)

**Keywords:** VT Re-ID, dual-path attention, cross-modality dual-constraint loss, local feature

## Abstract

Visible thermal person re-identification (VT Re-ID) is the task of matching pedestrian images collected by thermal and visible light cameras. The two main challenges presented by VT Re-ID are the intra-class variation between pedestrian images and the cross-modality difference between visible and thermal images. Existing works have principally focused on local representation through cross-modality feature distribution, but ignore the internal connection of the local features of pedestrian body parts. Therefore, this paper proposes a dual-path attention network model to establish the spatial dependency relationship between the local features of the pedestrian feature map and to effectively enhance the feature extraction. Meanwhile, we propose cross-modality dual-constraint loss, which adds the center and boundary constraints for each class distribution in the embedding space to promote compactness within the class and enhance the separability between classes. Our experimental results show that our proposed approach has advantages over the state-of-the-art methods on the two public datasets SYSU-MM01 and RegDB. The result for the SYSU-MM01 is Rank-1/mAP 57.74%/54.35%, and the result for the RegDB is Rank-1/mAP 76.07%/69.43%.

## 1. Introduction

Person re-identification (Re-ID) is a matching task, the purpose of which is to retrieve a specific person from multiple cameras placed in different positions [1,2,3,4,5]. Due to safety considerations and the increasing number of surveillance cameras in some public places, person re-identification plays an important role in intelligent video surveillance. However, in a 24-h intelligent monitoring scenario, a visible light camera alone cannot capture a clear image of a pedestrian. Therefore, a thermal camera needs to be used to collect a thermal image of a pedestrian, and the thermal image needs to match the visible light image. Thus, cross-modality person re-recognition [6,7,8,9,10], besides the differences within the modality, such as viewing angle changes, posture changes, etc., also need to consider the differences between modality. Therefore, cross-modality person re-identification is a challenge.

Firstly, observing the local features of different positions on the pedestrian image, there is an inherent relationship with the image feature description. For example, the relative positions of the pedestrian’s arms and legs are fixed, and there is an inherent structural relationship. However, the traditional CNN-based methods [11,12,13,14,15] use convolution kernels to extract image features sequentially. It can only perform feature extraction on locally related information, and cannot extract the correlation between features at different locations. Therefore, we introduced the attention mechanism module [16,17,18,19,20] to capture the spatial position information between each position of the pedestrian feature map. For a feature at a certain position, the map is updated via aggregating features at all positions with the weighted summation, in which the weights are determined by the degree of the internal relationship between the corresponding two positions. That is, any two positions with internal relationships can contribute to each other regardless of their distance in the spatial dimension, thus enhancing their ability to express characteristics. This relationship can effectively align the local features of the pedestrians in the two modalities and help improve visible thermal pedestrian re-identification.

Secondly, visible thermal person re-identification is based on the traditional triplet-base method. It focuses on the mining of triplet-wise samples, but the selection of triplet samples causes the data distribution to not necessarily be uniform, so the model training process is very unstable, the convergence is slow, and it is easy to overfit. Therefore, we propose a cross-modality dual-constraint loss. First, we add a center constraint for each class distribution by setting a center sample comparison manner. It pulls all samples of every class to the center of the corresponding class, which may effectively modify the intra-class relationship, reduce the changes among the category, and promote the aggregation of the class. Second, our loss function also adds boundary constraints by setting an upper bound for a positive sample pair and a lower bound for a negative sample pair. Restricting the boundary of the positive and negative sample pairs could further promote intra-class compactness, enhance inter-class separation, and reduce the cross-modality discrepancy.

Our framework has two main steps: firstly, we design a dual-path attention network model, which aims to extract richer features by focusing on contextual dependencies at arbitrary locations from a global perspective during modality-specific feature extraction. Secondly, to enhance the discrimination of features, we propose cross-modality dual-constraint loss, which uses central and boundary constraints for restrictions to promote intra-class compactness and enhance inter-class separability, therefore reducing inter-modal differences.

The contributions of this paper can be summarized as follows:

(1) We introduce a dual-path attention mechanism network model to focus on the spatial correlation between any two local features.
(2) We propose a cross-modal dual-constraint loss function to constrain the center of classes and the boundary, making the intra-class compact and the inter-class separable
(3) Our approach achieves good performance on RegDB and SYSU-MM01 datasets and performs favorably against existing methods

The rest of this paper is organized as follows: Section 2 provides related work. Section 3 provides our approach. Experiments are presented in Section 4. Finally, Section 5 concludes this work.

## 2. Related Work

Most existing Re-ID works are designed for single visible modality, where person images are captured by visible cameras. Current Re-ID methods have two key points: one is feature extraction, where the target pedestrian image and the candidate pedestrian image are used to extract robust pedestrian features; the other is metric learning, where the distance between the two feature vectors is calculated and their similarity is compared. Earlier works mainly used color histograms, Gabor features [1], HOG features [3], etc., to manually extract pedestrian features, and then used LMNN [21], PRDC [22], or other algorithms for similarity metric learning. Recently, deep learning has achieved increasing attention due to its superior performance. A detailed overview about Re-ID in single visible modality can be found in [2]. Most of these techniques developed for the single visible field are not suitable for the cross-modality pedestrian re-identification problem [23].

VT Re-ID is a research direction that has been emerging in recent years, mainly focused on the matching between thermal images and visible images, different from traditional pedestrian re-recognition technology which focuses on the matching between visible images and other visible images. Therefore, there are two major problems in cross-modality pedestrian re-recognition: inter-modality differences and intra-modality differences. Therefore, many methods have been proposed to solve the above problems. Ye et al. [24] learned the common features of visible and thermal images by using dual-path network structure, and proposed fusion feature loss and contrast loss for similarity learning. Dai et al. [25] applied GAN to cross-modal pedestrian re-recognition for the first time, and proposed a cross-modal generative adversarial network, which uses generators to learn features in different modes. Wang et al. [8] proposed a two-stage difference reduction method, which uses GAN to generate visible (infrared) images in order to generate their corresponding infrared (visible) images, ultimately forming a unified multispectral image. Liu et al. [26] proposed an enhanced discriminant feature learning method, which adopts the end-to-end dual-flow network structure and integrates the species layer features to extract more robust features. Zhang et al. [27] designed a dual-path space structure preserving public space network (DSCSN) and a contrast network (CCN), which used three-dimensional tensors to represent feature spaces to increase the learning of contrast features. Basaran et al. [28] proposed the distinguishing features of four-stream network structure learning. Image features were converted as input, and CNN was used for training in each stream to learn different and complementary features. Wang et al. [29] proposed generating cross-modal paired images and performing global ensemble-level and fine-grained instance-level alignment, which can perform ensemble-level alignment by unraveling mode-specific and mode-invariant features, while generating cross-modal paired images from exchanged images and minimizing the distance between each pair of images to perform instance-level alignment directly.

In addition to the unified feature model and modal transformation methods mentioned above, attention mechanisms and metric learning are also used for cross-modal pedestrian re-recognition tasks. The attention helps to focus people’s attention on the key part of the image, and extract important information from the key part of the feature. Hu et al. [16] proposed Squeeze-and-Excitation Networks (SENet), in which the attention mechanism can be used to correct features. After correction, valuable features are retained and worthless features are eliminated. The main contribution of the non-local neural networks proposed by Wang et al. [17] is to focus on long-term relationships. Stollenga et al. [19] proposed a Deep Attention Selective Network (DasNet), which dynamically changes the attention mechanism through reinforcement learning after training. Elsayed et al. [30] proposed that Saccader is a novel hard attention module. The key is the pre-training step, which only requires the class label and provides the initial attention position for gradient optimization. Cao et al. [18] combined SENet and non-local neural networks to obtain a GCNet network model to obtain global upper and lower information.

Metric learning, also known as similarity learning, enables the model to stay close to similar samples and far from different samples. Ye et al. [31] considered both inter-modality and intra-modality changes, and designed a high-order loss constraint based on bidirectional constraints to constrain pedestrian features on the basis of a two-path network structure. In order to reduce the burden of the network, Zang et al. [32] proposed a general multipartite network, in which these branches cooperate in learning to deal with different scenarios. Zhu et al. [6] proposed that the loss of the heterogeneous center can reduce intra-class transmorphological changes. Zhao et al. [33] designed a new hard five-state loss combined with characteristic loss and transplanted the recognition network used for single mode to cross mode. Hao et al. [24] proposed the hypersphere popular embedding network, which combined identity loss and ranking loss training models by mapping the learned shared features onto the hypersphere. Ye et al. [34] proposed a pattern aware collaborative learning method based on a dual-flow network to deal with modal differences, and proposed a collaborative learning scheme to standardize the identity classifier. Liu et al. [10] proposed centering triplet loss to reduce the strict constraint of triple loss by comparing the anchor center with all other centers instead of anchors with all samples.

## 3. Our Approach

**Figure 1:**
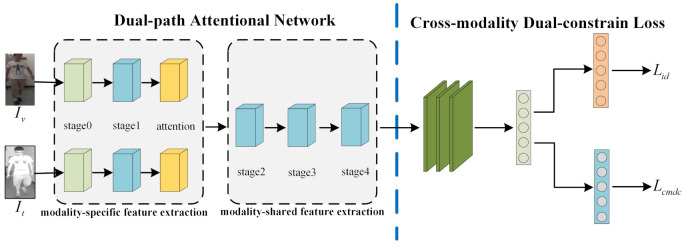
The pipeline of our proposed method for cross-modality person Re-ID contains two components: a dual-path attention network and cross-modality dual-constraint loss. The network consists of two stages. In the first stage, the modality-specific feature extraction is used to learn specific features of the visible and thermal modalities. In the second stage, the modality-shared feature extraction is used to learn the common features between the two modalities. The experiment has two constraints: (1) cross-modality dual-constraint (CMDC) loss; (2) identity (ID) loss.

In this section, we introduce our designed cross-modality person re-identification feature learning framework, as shown in Figure 1. The framework is mainly composed of two parts: (1) dual-path attention network and (2) cross-modality dual-constraint loss.

### 3.1. Dual-Path Attention Network

The dual-path network is a conventional way to extract features in visible thermal person re-identification, first introduced in [35]. It is composed of modality-specific feature extraction and modality-shared feature extraction. The modality-specific feature extraction aims to learn modality-specific information from visible and thermal modalities, while the modality-shared feature extraction focuses on learning the modality-shared features for cross-modality re-identification by projecting those modality-specific features into a modality-shared common feature space. In this situation, two problems warrant attention:

(1) Modality-specific feature extraction consists of two branches that do not share the parameters. If each branch contains an entire CNN architecture, the number of network parameters will increase exponentially.
(2) The internal connection between local features is neglected in the modality-specific feature extraction process.

In order to deal with the above two issues, we adopt the ResNet50 model as the backbone, with the consideration of its modular structure to reduce the design space of the network, and the bottleneck layer in the module can reduce the amount of calculation. The RestNet50 model consists of one shallow convolution block, stage0, and four res-convolution blocks. To split the RestNet50 model into our network, we use the shallow convolution block, stage0, and the first block, stage1, as the modality-specific feature extraction part, and stage2, stage3, and stage4 as the modality-shared feature extraction part.

**Figure 2:**
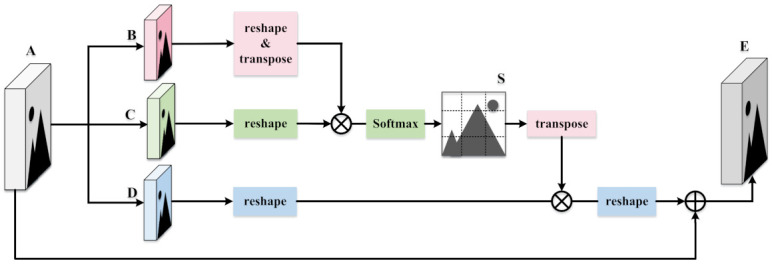
The details of the cross-modality attention module.

In addition, to extract better features when extracting specific features of the modality, we use the cross-modality attention module to achieve a more meaningful description of all position features. The cross-modality attention block captures the spatial correlation of any position of the pedestrian feature map, establishes rich contextual dependencies, and thus encodes the transformation of context information into local information. Next, we elaborate on the process of cross-modality attention, as is shown in Figure 2.

First of all, given a feature $A\in R^{C\times H\times W}$, we first feed it into convolution layers to generate two feature maps B and C, where $\left\{B,C\right\}\in R^{C\times H\times W}$. Then, we reshape them to $R^{C\times N}$, where N=H×W is the number of pixels. After that, we perform a matrix multiplication between the transposition of B and C, and work through the softmax layer to compute the spatial attention map $S\in R^{N\times N}$, where each element of the S matrix is:

(1) $$S_{ji}=\frac{exp\left(B_{i}\cdot C_{j}\right)}{\sum_{i=1}^{N}exp\left(B_{i}\cdot C_{j}\right)}$$

where $S_{ji}$ represents the effect of position ith on position jth. A higher degree of similarity between the feature representations of the two positions contributes to a greater correlation between them.

Meanwhile, we feed A into a convolution layer to generate a new feature map $D\in R^{C\times H\times W}$ and reshape it to $R^{C\times N}$. Then, we perform a matrix multiplication between the transposition of S and D and reshape the result to $R^{C\times H\times W}$. Finally, we multiply it by a scale parameter α and perform an element-wise sum operation with the feature A to obtain the output $E\in R^{C\times H\times W}$ as follows:

(2) $$E_{j}=\alpha \sum_{n=1}^{N}\left(S_{ji}\cdot D_{i}\right)+A_{j}$$

where α is a scale parameter, initialized as 0 and gradually taught to assign more weight. The result $E_{j}$ at each position is a weighted sum of the features across all positions and original features. $D_{i}$ is the element of D, and $A_{j}$ is the element of A.

In particular, S is equivalent to attention. Each line calculates the dependence between all pixels and a certain pixel. Softmax is probabilistic: the larger the value of softmax, the more reliable and the stronger the relative dependency.

Therefore, it has the information of global context and the selectivity of aggregating context according to the spatial attention map to extract better features. In addition, the cross-modality attention network has been proven to play an important role in VT Re-ID tasks.

### 3.2. Cross-Modality Dual-Constraint Loss

In this section, we introduce the designed cross-modality dual-constraint loss to guide network training for feature learning. The learning objective is to deal with both cross-modality discrepancy and intra-modality variations.

Triplet loss, which is one of the widely used methods for metric learning, helps to enhance the ability of feature discrimination. However, the selection of triplets causes the distribution of data to not necessarily be uniform, so the performance of the model training process is very unstable, and the convergence is slow. It is essential to continuously alter the parameters in line with the results, and the triplet loss is easier to overfit. Thus, these traditional methods are not well-applicable to cross-modality images.

Therefore, we propose a cross-modality dual-constraint loss. Firstly, we set the center constraint, which aims to provide a class center for each class, minimizing the distance between each sample and the corresponding class center, so that the purpose of reducing the distance within the class will be achieved. The details of the center constraint are as follows:

(3) $$\begin{array}{ccc} L_{c} & = & -\sum_{i=1}^{m}log\frac{e^{W_{y_{i}}^{T}x_{i}+b_{y_{i}}}}{\sum_{j=1}^{n}e^{W_{j}^{T}x_{i}+b_{j}}}+\frac{1}{2}\sum_{i=1}^{K}D\left(x_{i},c_{y_{j}}\right)\\ \; & = & -\sum_{i=1}^{m}log\frac{e^{W_{y_{i}}^{T}x_{i}+b_{y_{i}}}}{\sum_{j=1}^{n}e^{W_{j}^{T}x_{i}+b_{j}}}+\frac{1}{2}\sum_{i=1}^{K}{∥x_{i}-c_{y_{i}}∥}_{2}^{2} \end{array}$$

where $x_{i}\in R^{d}$ denotes the ith deep feature, belonging to the $y_{i}\mathrm{th}$ class; $W_{j}\in R^{d}$ denotes the $y_{i}\mathrm{th}$ column of the weights $W\in R^{d\times n}$ in the last fully connected layer; and $c_{y_{i}}\in R^{d}$ denotes the ith class center of deep features. The formulation effectively characterizes the intra-class variations and inter-class changes.

Secondly, we set the boundary constraints, which take the form of a distance metric on the space of shapes, and use integrals over the boundary between the regions. Furthermore, it provides information that is complementary to the center constraint. Under these circumstances, we can effectively suppress negative pairs far away from the border area, eliminate outliers far away from the border area, and all positive and negative samples are bounded. Finally, expressed mathematically, our cross-modality dual-constraints loss is formulated by:

(4) $$L_{cmdc}=\sum_{i=1}^{K}\left(maxD\left(P_{i},C_{i}\right)-minD\left(C_{i},C_{j}\right)+\alpha \right)$$

where $C_{i}$ is the center of $P_{i}$, which could be the thermal or visible feature for ith class; and K is the total number of identity classes. We use a marginal threshold α between the distance of $P_{i}$ and $C_{i}$ versus $C_{i}$ and $C_{j}$. The $maxD\left(P_{i},C_{i}\right)$ is the upper bound of the distance of positive pairs, and $minD\left(C_{i},C_{j}\right)$ is the lowest bound of the distance of negative pairs. Different from other methods, our CMDC compares the class center and class sample, and pushes the boundary of the two sets, while other methods give a comparison between samples, pushing each sample apart in positive and negative pairs. With the training epoch growing, there is a clear dividing line between positive pairs and negative pairs in feature embedding space. Hence, the learned features belong to the same class close to each other, while those of different classes are far away from each other.

In addition, to achieve better classification results, we also take identity loss into consideration, which integrates identity-specific information by treating each person as one class. The identity loss is also added to the model training to enhance the robustness of the feature learning process. The formula for identity loss is given as follows:

(5) $$\begin{array}{c} L_{id}=\sum_{i=1}^{N}-q_{i}log\left(p_{i}\right)\\ \begin{array}{cc} s.t. & q_{i}=\left\{\begin{array}{cc} 1-\frac{N-1}{N}\xi , & y=i,\\ \;\;\;\;\;\frac{\xi }{N}, & y\neq i, \end{array}\right. \end{array} \end{array}$$

where $p_{i}$ is the ID prediction logits of the ith class, *N* is the number of identities in the total training set, and ξ is a constant, for the purpose of improving the training.

The total loss function is the weighted sum of identity loss $L_{id}$ and our loss function $L_{cmdc}$:

(6) $$L=L_{id}+\lambda L_{cmdc}$$

where λ is the weight parameter to balance the importance of our loss function.

In summary, our CMDC has the following advantages compared with other loss functions. Firstly, CMDC not only replaces the comparison between samples by setting the class center, but also considers the boundary constraints between positive and negative pairs. Secondly, it also has great advantages for mining difficult samples. Finally, we think our CMDC is easy to implement and may be combined with other methods.

## 4. Experiment

**Figure 3:**
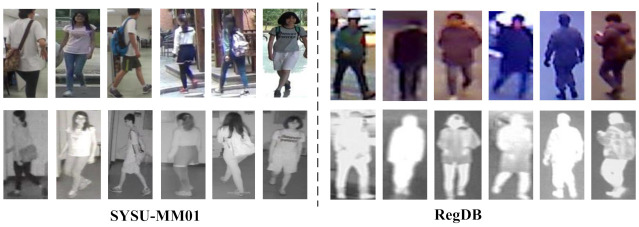
Example pictures from SYSU-MM01 and RegDB. The pictures in the first component are from the SYSU-MM01, in which the first row is the pictures captured by a visible camera and the second row is the images captured by a thermal camera. The same is true for RegDB. Each column includes pictures of the same person.

In this section, we test the effectiveness of our proposed approach on two public datasets, SYSU-MM01 [23] and RegDB [36]. The example pictures are shown in Figure 3.

### 4.1. Experimental Settings

#### 4.1.1. Datasets and Settings

SYSU-MM01 is a public large-scale dataset of VT Re-ID. It is collected through six cameras (four visible and two thermal). Some cameras are deployed indoors, others outdoors. The dataset contains 491 pedestrians with different identities, of which 296 are used for training, 99 are used for verification, and 96 are used for testing. There are 30,071 RGB images and 15,792 thermal images in total. In the all-search mode, the image contains all of the visible images collected by the four visible cameras; in the indoor-search mode, the images only contain the visible images collected by the two indoor visible cameras. The all-search mode is more demanding than the indoor-search mode.

RegDB is captured by two aligned cameras (one visible and one thermal) and contains 412 pedestrian identities. For each pedestrian, 10 RGB images and 10 thermal images are collected, of which there are 254 women and 158 men, and of the 412 people, 156 were taken from the front and 256 were taken from the back. Furthermore, the dataset is randomly divided into two halves, one for training and the other for testing. This process was repeated 10 times to obtain a statistically stable result, and the average value was recorded.

#### 4.1.2. Evaluation Metrics

Following existing works, cumulative matching characteristics (CMC) and mean average precision (mAP) are adopted as the evaluation metrics. CMC (Rank-r accuracy) measures the probability of a correct cross-modality person image occurring in the top-r retrieved results. mAP measures the retrieval performance when multiple matching images occur in the gallery set.

#### 4.1.3. Implementation Details

Our algorithm is implemented with PyTorch on NVIDIA RTX 2080Ti. We choose RestNet50 as the backbone network. The input pictures used for training and testing are adjusted to 288×144 by random cropping with zero padding and horizontal flipping. We set the initial learning rate as 0.1 for training with the epoch 60, batch size 8. The learning rate will continue to update with the number of iterations. We use stochastic gradient descent (SGD) as an optimizer for optimization, and the momentum parameter is set to 0.9.

### 4.2. Comparison with the State-of-the-Art

As is proven in Table 1, we evaluate our approach with state-of-the-art methods on the SYSU-MM01 dataset, including HCML [35], BDTR [31], DRL [8], MAC [34], AlignGAN [37], CMSP [38], AGW [39], DFE [40], XIV [41], DDAG [42], HAT [43], and NFS [44]. The outcomes show that our proposed approach can achieve a comparable performance to that of HAT, being 2.45% and 0.46% greater than Rank-1 and mAP, and 1.61% and 1.09% less than Rank-10 and Rank-20. The HAT method will artificially increase the quantity of data through data expansion; therefore, it leads to the Rank-10 and Rank-20 indicators. Our method uses the original dataset and does not use the strategy of data expansion. Therefore, although fewer datasets are used, the effect is still very good. In addition, compared with the NFS, we are 0.83% ahead on Rank-1; and 0.81%, 0.25%, and 1.1% behind on Rank-10, Rank-20, and mAP. The NFS method performs feature extraction on both coarse-grained and fine-grained channels simultaneously, with high time complexity. Our method only adds the attention module based on the backbone, which has the advantages of low time complexity, being lightweight, and excellent Rank-1 performance.

On the other hand, we also document the evaluation on RegDB to confirm the superiority of the proposed approach. As is shown in Table 2, the proposed approach achieves a superior performance compared with peer works in terms of most metrics. Notably, our approach compares favorably against AGW and DDAG. Our approach surpasses HAT and DFE, with 4.24% and 5.94% in Rank-1, respectively. In terms of other previous works, such as AlignGAN, our approach outperforms them by a large margin: over 18% and 15% improvement according to Rank-1 and mAP, respectively.

The experimental results on two datasets show that our proposed approach achieved good results on both small-scale and large-scale datasets.

### 4.3. Ablation Experiments

We evaluate the effectiveness of the dual-path attention network (DPAN) module and cross-modality dual-constraint (CMDC) loss on SYSU-MM01, as shown in Table 3. The setting of using only the DPAN module provides 3.75%, 2.09%, and 2.25% improvements over the baseline for Rank-1, Rank-10, and mAP, respectively, which indicates the necessity of establishing rich contextual relationships in cross-modality person Re-ID. The setting of using only CMDC loss provides 3.34%, 1.39%, and 1.64% improvements over the baseline for Rank-1, Rank-10, and mAP, respectively, which indicates the necessity of setting central and boundary constraints for restrictions in cross-modality person Re-ID. Moreover, compared with our baseline, the “Baseline + DPAN + CMDCLoss” setting achieves the best performance across all evaluation metrics, achieving 6.39%, 4.73%, and 4.87% improvements over the baseline for Rank-1, Rank-10, and mAP, respectively.

### 4.4. Parameter Analysis

**Figure 4:**
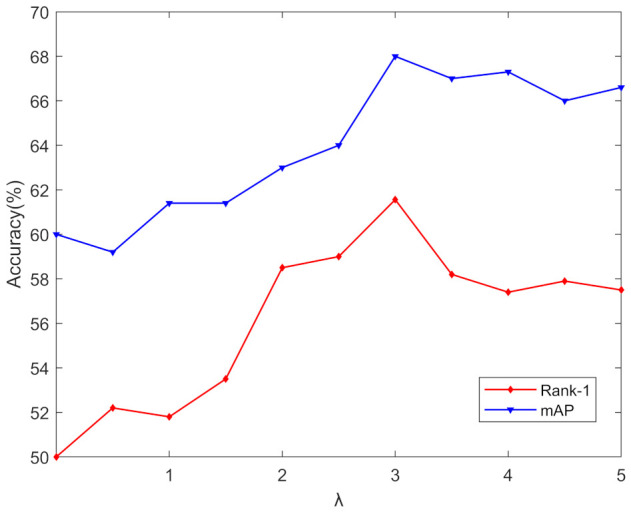
Influence of λ on SYSU-MM01 dataset. λ is the balance weight of $L_{cmdc}$.

**Figure 5:**
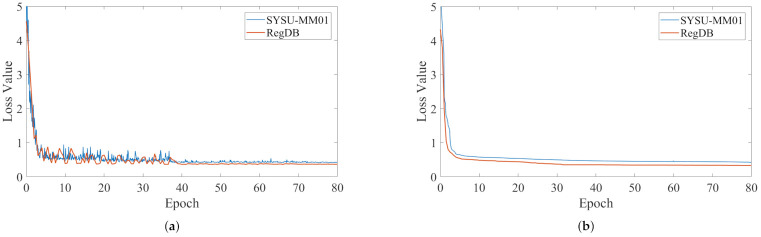
Convergence curve of CMDC on SYSU-MM01 and the RegDB dataset. (**a**) Training convergence curve of CMDC. (**b**) Validation convergence curve of CMDC.

This subsection assesses the significance of our loss. By adjusting different balance parameters, we produce results concerning $L_{cmdc}$, as shown in Figure 4. As λ increases, the accuracy improves first. When λ = 3, our approach achieves high-quality overall performance, due to our use of the Euclidean metric. When its value becomes larger than reasonable, it dominates the total training loss, which is detrimental to training and may lead to divergence. Therefore, as λ increases further, it can damage the feature learning process, and the performance drops dramatically. In addition, the convergence curve of the cross-modality dual-constraint loss can be seen in Figure 5. Empirically, we can see that the objective function value drops quickly and becomes stable after several iterations.

## 5. Conclusions

In this paper, we propose a dual-path attention network for cross-modality person re-identification. The network structure extracts modality-specific information and modality-shared information. When extracting modality-specific information, attention is paid to the internal relationships between local features, establishing rich contextual relationships. At the same time, to guide the training process, a cross-modality dual-constraint loss function is introduced to promote intra-class compactness and enhance the separability of the inter-class. The proposed loss function takes the intra-modality and inter-modality changes and ensures taking the discrimination of cross-modality person re-identification learning features into consideration. Many experiments carried out on SYSU-MM01 and RegDB show that our method has advantages over existing methods.

## Figures and Tables

**Table 1:** Comparison with the state-of-the-art method on the SYSU-MM01 dataset. Re-identification rates at Rank-r and mAP.

Method	Source	All Research				Indoor Research			
		Rank-1	Rank-10	Rank-20	mAP	Rank-1	Rank-10	Rank-20	mAP
HCML	AAAI18	14.32	53.16	69.17	16.16	24.52	73.25	86.73	30.08
BDTR	IJCAI18	17.00	55.40	69.20	16.20	-	-	-	-
DRL	CVPR19	28.90	70.60	82.40	29.20	31.60	77.20	89.20	44.20
MAC	MM19	33.26	79.04	90.09	36.22	36.43	63.36	71.63	37.03
AlignGAN	ICCV19	42.40	85.00	93.70	40.70	45.90	87.60	94.40	54.30
CMSP	IJCV20	43.56	86.25	-	44.98	48.62	89.50	-	57.50
AGW	Arxiv20	47.50	-	-	47.65	54.17	-	-	62.97
DFE	MM19	48.71	88.86	95.27	48.59	52.25	89.86	95.85	59.68
XIV	AAAI20	49.92	89.79	95.96	50.73	-	-	-	-
DDAG	ECCV20	54.75	90.39	95.81	53.02	61.02	94.06	98.41	67.98
HAT	TIFS20	55.29	**92.14**	**97.36**	53.89	62.10	95.75	99.20	69.37
NFS	CVPR21	56.91	91.34	96.52	**55.45**	**62.79**	**96.53**	**99.07**	**69.69**
Our		**57.74**	90.53	96.27	54.35	61.56	94.86	98.34	68.13

**Table 2:** Comparison with the state-of-the-art methods on RegDB dataset in visible → thermal.

Method	Source	Rank-1	Rank-10	Rank-20	mAP
HCML	AAAI18	24.44	47.53	56.78	20.80
BDTR	IJCAI18	33.56	58.61	67.43	32.76
DRL	CVPR19	43.40	66.10	76.30	44.10
MAC	MM19	36.43	62.36	71.63	44.10
AlignGAN	ICCV19	57.90	-	-	53.60
XIV	AAAI20	62.21	83.13	91.72	60.18
CMSP	IJCV20	65.07	83.71	-	64.50
DDAG	ECCV20	69.34	86.19	91.49	63.46
AGW	Arxiv20	70.05	-	-	66.37
DFE	MM19	70.13	86.32	91.96	67.56
HAT	TIFS20	71.83	87.16	92.16	67.56
Our		**76.07**	**90.44**	**93.98**	**69.43**

**Table 3:** Evaluation of each component on SYSU-MM01.

Setting	Rank-1	Rank-10	mAP
Baseline	51.35	85.80	49.48
Baseline + DPAN	55.10	87.89	51.73
Baseline + CMDC	54.69	87.19	51.12
Baseline + DCAN + CMDC	**57.74**	**90.53**	**54.35**

## Data Availability

Not applicable.
